# Imaging Modality to Guide Left Atrial Appendage Closure: Current Status and Future Perspectives

**DOI:** 10.3390/jcm12113756

**Published:** 2023-05-30

**Authors:** Giulia Laterra, Giuseppe Dattilo, Michele Correale, Natale Daniele Brunetti, Claudia Artale, Giorgio Sacchetta, Lorenzo Pistelli, Marco Borgi, Francesca Campanella, Federica Cocuzza, Maria Claudia Lo Nigro, Marco Contarini

**Affiliations:** 1 Cardiology Unit, Department of Emergency, Umberto Primo Hospital, 94100 Enna, Italy; giulia.laterra1990@gmail.com; 2 Section of Cardiology, Department of Biomedical and Dental Sciences and Morphofunctional Imaging, University of Messina, 98122 Messina, Italy; dattimed@hotmail.it (G.D.); pis.lorenz@gmail.com (L.P.); marco.borgi9@gmail.com (M.B.); campanellafrancesca95@gmail.com (F.C.); federica.cocuzza3@gmail.com (F.C.); mariaclaudialonigro@gmail.com (M.C.L.N.); 3 Cardiothoracic Department, Policlinico Riuniti University Hospital, 71122 Foggia, Italy; natale.brunetti@unifg.it; 4 Cardiology Unit, Department of Emergency, Umberto Primo Hospital, 96100 Siracusa, Italy; claudia.artale@gmail.com (C.A.); giorgiosacchetta@gmail.com (G.S.); marcocontarini@gmail.com (M.C.)

**Keywords:** left atrial appendage (LAA), atrial fibrillation, intracardiac echocardiography, NOAC

## Abstract

Atrial fibrillation (AF) is the most common cardiac arrhythmia in adults. The left atrial appendage (LAA) is the most likely source of thrombus formation in patients with non-valvular atrial fibrillation (NVAF). Left atrial appendage closure (LAAC) represents an effective alternative to NOAC in patients with NVAF. Expert consensus documents recommend intraprocedural imaging by means of either transesophageal echocardiography (TEE) or intracardiac echocardiography (ICE) in addition to standard fluoroscopy to guide LAAC. TEE-guided LAAC usually requires general anesthesia. The ICE technique is a “minimalist approach”, without general anesthesia, but ICE imaging techniques are not yet simplified and standardize, and the ICE may result in inferior image quality compared with that of TEE. Another “minimalist approach” can be the use of ICE via the esophageal route (ICE-TEE), that jet is validated to identify the presence of LAA thrombi in patients and to perform other procedures. In our cath laboratory ICE-TEE to guide LAAC is used in some complex patients. Indeed, our single center experience suggests that ICE-TEE could be a good alternative imaging technique to guide LAAC procedure without general anesthesia.

## 1. Introduction

Atrial fibrillation (AF) is the most common cardiac arrhythmia in adults. The prevalence of AF in adults is between 2% and 4%. This percentage significantly increases with age [1], lifetime AF risk estimate of 1 in 3 individuals of European ancestry at an index age of 55 years [2,3].

In the treatment of AF patients, together with achieving better symptom control and treating comorbidities, avoiding stroke is of paramount importance, as its consequences are often dramatic [4].

Perhaps, AF has been shown to increase the risk of stroke five-fold; furthermore, AF-related stroke is generally more severe when compared to non-AF-related stroke causing greater disability or worse outcome. Whereas symptomatic burden and AF pattern (Paroxysmal, Persistent, long-standing Persistent, Permanent) are crucial in deciding the anti-arrhythmic strategy, stroke risk has been shown to be affected by the presence of specific risk factors and not by AF patterns, CHA2DS2-VASc is the score most used in clinical practice to identify patients at high stroke risk and to define those who might benefit from oral anticoagulation, evaluating the presence of congestive heart failure, hypertension, age, diabetes mellitus, previous stroke, vascular disease, and female sex [5,6,7,8,9,10].

In order to prevent cerebral ischemic events, oral anticoagulation (OAC) is currently indicated as first-line therapy in patients with non-valvular AF and CHA_2_DS_2_-VASc score ≥2 (3 in females). The implementation of direct oral anticoagulants (DOAC) provided an improvement in the field, as these drugs were proved to be safer and more effective with respect to vitamin-K antagonists (VKA) [11], as bleeding still represents a major issue in a wide category of patients, new drugs are under development with the hope to achieve effective stroke protection without increasing the bleeding risk [3,12].

Unfortunately, anticoagulation is not feasible in a non-negligible number of patients, and often adherence to treatment is poor, hence providing suboptimal protection from ischemic stroke. A recently published meta-analysis enrolled 594 784 AF patients currently on DOAC, showing acceptable adherence in 66% of patients (95% C.I. 63–70%) and acceptable persistence in 69% (95% C.I.; 65–72%), with non-significant differences among different agents; such a low adherence and persistence in 1 out of 3 patients was associated with poor clinical outcomes, and represents an issue to be taken into consideration while deciding an anti-thrombotic strategy [13]. As a matter of fact, patients with relative or absolute contraindications to OAC, with high bleeding risk under chronic OAC, or unwilling to assume medical therapy constitute a clinical conundrum and still pose a difficult clinical dilemma to the treating physician.

It is widely accepted the concept that the left atrial appendage (LAA) represents the most likely site of thrombus formation in NVAF patients [3,12,13,14,15,16,17,18]. Indeed, a meta-analysis by Mahajan et al. demonstrated that 89% of thrombi in the left atrium (LA) were located in the LAA [19], and Blackshear et al. demonstrated that thrombus was present in about 17% of patients with non-valvular AF and of these, in 91% of cases it was localized in LAA [20].

Such a thorough understanding of the pathophysiology related to thrombus formation led researchers to the development of new techniques to perform an alternative stroke prevention therapy left atrial appendage closure (LAAC). Different devices have hence been designed and developed to perform the LAAC procedure. Watchman and its evolution Watchman FLX, by Boston Scientific, and Amulet Amplatzer, by Abbott, have been the first devices to report several studies demonstrating their safety and effectiveness.

Two large RCTs, PROTECT-AF [21] and PREVAIL [22], enrolled 1114 patients with NVAF, comparing LAAC to OAC with VKA, showing non-inferiority of the interventional treatment with respect to the medical option, a procedure that proved to be safe and effective. The meta-analysis of the two trials showed The WATCHMAN device was non-inferior to warfarin for the composite of stroke, systemic embolism, and cardiovascular death, with better outcomes in terms of mortality, hemorrhagic stroke, and major bleeding, with a higher number of ischemic stroke in the WATCHMAN group not reaching statistical significatively [23].

After FDA approval, implantation rates all over the world kept growing as LAAC did show to meet a very important clinical need, and implant procedure proved to be safe in real-world; in one of the largest nationwide US registries enrolling 3822 consecutive patients, data were better when compared to those reported in registrational studies with an implant success rate as high as 95.6% with a low complication rate (1.5%). The introduction of new imaging techniques certainly contributed to the general improvement in technical success, both with pre-procedural planning and peri-procedural guidance [24].

As warfarin is nowadays rarely used in clinical practice, with DOAC being preferred in most cases, PRAGUE-17 trial compared WATCHMAN or AMULET device to DOAC, mostly apixaban, showing non-inferiority in terms of cardiovascular death, stroke/transient ischemic attack, systemic embolism, clinically significant bleeding (ISTH major, or nonmajor clinically significant bleeding), or significant procedure/device-related complication (hazard ratio [HR] 0.84, *p* for noninferiority = 0.004) with long term results (3.5 years of follow-up) showing statistically significant superiority of LAAC compared to DOAC with respect to clinically relevant bleeding (HR 0.55, *p* for superiority 0.039) [21]. Among the most important complications, certainly, we should take into account device-related thrombus (DRT); in a meta-analysis enrolling over 10,000 patients who underwent LAAC, the incidence of DRT was estimated to be 3.7% with the first-generation devices; in a recent prospective study including 301 patients with the use of second-generation device WATCHMAN-FLX incidence of DRT was 1.7% [25].

Among the many ongoing trials which are currently comparing NOAC with LAAC most relevant are OCCLUSION-AF (all LAAC devices), COMPARE LAAO, CATALYST (Amplatzer Amulet, Plymouth, MN, USA), and CHAMPION-AF (Watchman FLX, Maple Grove, MN, USA) [26,27,28,29,30,31,32,33,34]. Other devices are available in the market, such as Lambre (Shenzhen, China), Kardia (Mountain View, CA, USA), and Omega (Biel, Switzerland), providing further options to the interventional cardiologist with the aim of providing of refining device choice and improving outcomes.

## 2. Procedural Steps

Before starting the procedure, TEE imaging should be performed to search for the presence of LAA thrombus. A femoral venous access is established with a standard introducer sheath (double venous accesses if ICE was used). Then, trans-septal puncture (TSP) imaging-guided is performed using the long and the short axes of the interatrial septum. For most of the devices closure, the TSP should be located inferiorly and posteriorly. Next, a 0.35 in extra-stiff wire is placed within the left upper pulmonary vein (LUPV). The wire used has a flexible, spiral tip with a supportive body, which facilitates catheter exchanges with the access sheath. The access sheath and dilator are advanced over the stiff wire located in the LUPV. After the wire and dilator are removed, instead the access sheath should be carefully flushed with saline. Then, a pigtail catheter is introduced into the sheath, advanced into the LUPV, and next located in the LAA and connected to the manifold for contrast injections. LAA angiography is performed when the pigtail catheter is intubated in LAA deeply. Then, measures obtained by ICE/TEE imaging are compared with fluoroscopic measures. Indeed, the device size should be selected by combining values obtained by TEE/ICE and cineangiography. The pigtail catheter is removed, and the device is introduced in LAA. When device positioning is considered acceptable, a tug-test is performed. TEE/ICE and angiography are then repeated to assess device position, to rule out the presence of a peri-device leak and pericardial effusion, and to assess position with respect to different cardiac structures. If all criteria are met, then the device can be safely deployed. It is advisable to perform transthoracic echocardiography (TTE) prior to discharge, which might even be the same day of the procedure, to confirm the correct positioning of the device hence excluding embolization and to confirm the absence of pericardial effusion. After discharge, it is advisable to repeat a TEE or Coronary computed tomography angiography (CCTA) after 6 to 12 weeks in order to rule out the presence of device-related thrombus (DRT) and peri-device leaks (PDL) [35].

## 3. Fluoroscopy

**Figure 1:**
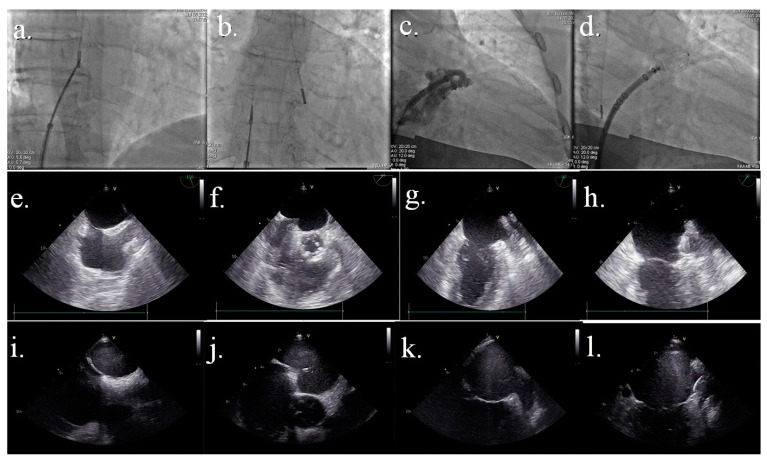
Comparison image between fluoroscopic, TEE, and ICE-TEE images. Figure 1a–d: fluoroscopic images. (**a**) transeptal step of the procedure with the catheter in the right atrium; (**b**) transeptal puncture performed with the catheter in the left atrium; (**c**) angiographic image to establish final LAA device size; (**d**) device deployed Figure 1e–h: TTE images. (**e**) bicaval view; (**f**) tenting of septal du-ring trans-septal puncture; (**g**) LAA view; (**h**) device successfully deployed Figure 1i–l: ICE-TEE images. (**i**) bicaval view; (**j**) tenting of septal during trans-septal puncture; (**k**) LAA view; (**l**) device successfully deployed.

Expert consensus documents recommend intraprocedural imaging by means of either TEE or ICE to guide LAAC. An all-comers LAAC registry demonstrated that the use of intraprocedural echocardiography to guide intervention in addition to standard fluoroscopy, compared with fluoroscopy alone, was independently associated with lower procedural complications and higher long-term technical success. Therefore, fluoroscopy is or was the only intraprocedural imaging modality used during LAAC at some centers. In these cases, following transseptal puncture (TSP), angiography of the LAA is performed with an injection of contrast in front of the LAA ostium using an RAO 20–30° with cranial angulation of 20° view. Then, angiography sizing of LAA is performed thanks to computerized quantitative coronary analysis (QCA). There were no significant differences in measurements of the maximal LAA ostium and landing zone measured by TEE vs. angiography. Though, TEE provides larger measurements as compared to angiography [35,36,37,38] (Figure 1a–d).

## 4. TEE

TEE has been the traditional gold-standard preprocedural imaging for LAAC, and currently, device-releasing criteria are based on TEE [35]. The key elements for preprocedural imaging are the exclusion of LAA thrombus, characterization of LAA anatomy, and the number of lobes and LAA measurements. Procedural imaging allows achieving optimal device implantation with adequate sealing of LAA, reducing the incidence of PDL and promptly diagnosing eventual complications, such as device embolization, pericardial effusion, and thrombus formation on sheaths, while performing the intervention, hence strongly contributing to increasing procedural safety, minimizing adverse events.

The advantages of TEE are it not requiring X-ray and contrast as compared to CCTA and being widely available in most centers for LAAC. Moreover, the evaluation with TEE can be carried out directly in the catheterization laboratory before starting the procedure avoiding an additional preprocedural imaging session. On the other hand, TEE is often associated with significant challenges, as general anesthesia constitutes a risk in many patients, especially if they are old and fragile; furthermore, TEE might cause damage to the esophageal mucosa, and it is contraindicated in patients affected by esophageal varices. The requirement of a dedicated anesthesiology team and a fully trained echocardiography certainly constitutes a logistic challenge, hence constituting another limitation to the use of TEE. As an alternative, CCTA provides accurate spatial resolution with optimal anatomical characterization and measurements when used as a pre-procedural imaging modality. TEE is still considered the gold standard for ruling out the presence of auricular thrombosis; as a matter of fact, newer CCTA protocols have made it feasible to exclude with sensitivity close to 100% the presence of thrombosis when CCTA shows good LAA contrast opacification, making TEE nonnecessary.

In addition to its role in pre-procedural planning, TEE is invaluably useful as a periprocedural tool for guiding the different LAAC procedural stages chose final sizes LAA after adequate LA pressure (>12 mmHg), guide transseptal puncture, guide catheter positioning, guide device positioning, and deployment, assess for pericardial effusion, assess surrounding structures and rule out the peri-device leak and device embolization. At the beginning of the procedure, it is important re-check the absence of LAA thrombus and next measure the ostium, landing zone, and length of the LAA, and the number of lobes using multiple views (0°, −45°, −90°, and 135° views). Successful device deployment depends on the ability to position the delivery sheath with sufficient depth into the LAA. The most favorable sheath orientation is obtained with a posterior transseptal puncture. For the Watchman device and the Amplatzer Cardiac Plug, an inferior-posterior transeptal puncture is recommended; instead, for the LARIAT device, a superior-posterior transeptal puncture is suggested. The TEE performs a crucial role in performing an optimal TSP: in fact, the inferior position and the posterior position are established using a long-axis 110° view of the septum and a short-axis 30° view of the septum, respectively.

The TEE enables monitoring every procedural step monitoring the position of the wire, pigtail, and sheath. Using this imaging technique, it is possible to check the correct device deployment using several criteria: position, anchor, size, and seal of the device (P.A.S.S. criteria). Anchor is assessed using tug test and compression rate. Ruling out the peri-device leak is another important evaluation before the final release of the device and must be performed in multiple views: 0°–45°–90°–135° with low Nyquist limit (<40 cm/s) [39,40,41,42,43] (Figure 1e–h).

Great effort is currently carried out to minimize discomfort to the patient, trying to develop smaller probes providing high-quality images, and more research is necessary on this topic.

## 5. Intracardiac Echography (ICE)

ICE has been used for many years to guide structural heart interventions, such as atrial septal defect and patent foramen oval closure, and many interventional electrophysiological procedures involving transeptal puncture. The feasibility and safety of LAA occlusion with ICE guidance have been proved recently. In many centers, it is currently performed as the standard approach for perioperative guidance, despite the need for additional vascular access (the increased risk of atrial septal defects, whether these have any clinical implication is yet to be determined), as it allows to carry out the procedure under local anesthesia with the patient awake and responsive. If no pre-procedural imaging is feasible, auricular thrombosis might be ruled out using distant contrast injection in LAA under fluoroscopy, with a non-negligible risk of thrombus embolization. For this reason, periprocedural ICE should always be supported by accurate pre-procedural planning using either TEE or CCTA in order to exclude the presence of auricular thrombosis and confirm correct device sizing measurements. Notably, LAA sizing obtained by the combined analysis of ICE images and angiography can slightly vary from measurements obtained with TEE and angiography, even though these differences might be relatively minor with no impact on overall clinical outcomes. Several studies have reported the feasibility and safety of ICE-guided LAAC using these approaches [44,45,46,47]. A recent meta-analysis compared ICE with TEE in guiding LAAC in 1157 patients showing no differences in acute procedural success among the two methods [RR 1.01 95% CI (0.99–1.04, *p* = 0.24)], with no difference in fluoroscopy time, total procedure time and complications including pericardial tamponade, device embolization and stroke between two groups [48]. In a recent multicenter prospective study enrolling 100 patients, no major intervention-related adverse events were reported at follow-up, with three vascular access bleeding reported, all resolved with no sequelae. Among the drawbacks of ICE, procedure and fluoroscopy times were quite long (median 50.5 min IQR 38.0–65.0 min) when compared to data using TEE. Same-day discharge was feasible with the ICE approach in a percentage of patients as high as 19%. Sizing was adequate, with mean device compression achieved of 19.2% ± 7.1% (recommended device compression rate for Watchman FLX 10–30%). Notably, preprocedural imaging was performed in all patients, either with TEE or CCTA, thus contributing to the achievement of an acceptable compression rate [49]. There are two categories of ICE catheters commercially available: rotational and phased-array catheters. The last one is steerable and offers far-field imaging, for which it is more used to guide structural heart disease procedures. Although the use of ICE has become widespread in cath-labs, still a widely accepted consensus document is lacking, and operators mostly rely on their own experience; such a lack of standardization certainly represents an obstacle for the young operator who approaches the method. Moreover, the complexity is increased by the variability in heart rotation that determines different imaged structures in the same catheter position. To perform LAAC using ICE, several approaches have been proposed. In the first, the ICE catheter is located in the right atrium (RA), but not in all patients’ LAA and the pulmonary veins can be seen. In the second, the transeptal puncture is guided by ICE in RA, and a stiff guide is located in LA, and over this guide catheter dilator is carried in LA. Subsequently, this dilatator is removed, and the ICE probe is carried in LA across the dilatate septum, close to the previous allocated wire [41]. The third approach involves two separate transeptal punctures. Regardless of the type of approach chosen, it is crucial that the ICE probe is left free to rotate clockwise or counter-clockwise and can be tilted to the left and to the right: such movements are crucial for proper visualization of the device in order to rule-out the presence of peri-device leaks using color Doppler. In addition to the previously listed movements, probe could also be advanced toward left upper pulmonary vein in order to obtain a long-axis view of LAA. Notably, additional transseptal crossing caused more residual iatrogenic atrial septal defects compared to TEE (35% vs. 26%) at the 45-day imaging in particular with the second approach described. Instead, device-related complications (device thrombus, embolization, and leak) at the follow-up are similar compared with TEE-guided LAAC [45] (Figure 1i–l).

As a matter of fact, there is a learning curve for ICE-guided LAAC, and this might slow down its use in some centers; in order to overcome such limitations, systematic training of operators, even with the use of dedicated simulators and with dedicated sessions at congresses, might help to let this important technology available in centers performing LAAC. Furthermore, using this technique to guide other procedures, such as septal defect or patent foramen oval closure, might speed the learning curve up allowing the operator to be confident with the methods.

Many efforts have been made to develop a new three-dimensional ICE probe in order to achieve better periprocedural guidance with a more accurate evaluation of sizing and of the landing zone, aiming to reduce both periprocedural and long-term adverse events. The use of a novel 3D-ICE probe has been reported, with the ability to provide tilting plane imaging together with 3D-color and spectral Doppler assessment, but currently, such devices are not approved for use in Europe, as they still have not obtained CE certification [50]. Clinical trials are needed in order to implement such an interesting technique in the future of interventional cardiology.

## 6. Future Perspectives

TEE-guided LAAC usually requires the administration of anesthetic drugs to manage the discomfort of the probe during the procedure and avoid patient movements. Patients referred for LAAC procedures are usually old, and they may have complications related to anesthesia (such as preoperative pulmonary complications, cognitive decline, etc.). This is the premise to identify a “minimalist approach”, without general anesthesia, in guiding LAAC. The ICE technique needs minimal sedation, and for this reason, it is the standard in some centers. Instead, the use of ICE via the esophageal route (ICE-TEE) is validated to identify the presence of LAA thrombi in patients with AF, and, when the LAA could be adequately imaged (around 85%), this technique has a sensitivity of 100% and a specificity of 97% [50].

ICE-TEE is concordant with standard TEE for the visualization of the LAA and atrial septal in the vast majority of patients. This alternative use of the ICE probe is safely used to monitor PFO closure without requiring general sedation [51]. Moreover, ICE-TEE is useful in detecting thrombus occurring after device implantation [52]. TEE using an intravascular ultra-sound catheter in rats experimented jet in 1997, and the safety was documented. In fact, in children or infants with oropharyngeal abnormalities, ICE-TEE was used to guide safely the repair of congenital heart disease [53]. Despite several studies highlighting the safety of ICE-TEE, this technique never has been used to guide LAAC. So, we have decided, after obtaining informed patient consent, to use ICE-TEE in guiding LAAC in our laboratory. The LAAC procedures were performed by operators with extensive experience in LAAC. At first, we have enrolled patients with oropharyngeal disease (narrowing, cancer, etc.), then complex patients in which general anesthesia and TEE carry a non-negligible risk of major complications. In these patients, we have performed the entire ICE-guided LAAC without anesthesia. Esophageal intubation for ICE-TEE has been successfully performed and well tolerated by the 10 patients (8 males, 72 ± 8 years-old, FE 54 ± 11%). A switch to standard approach with TEE was not required in any patients. The probe has been introduced in the mid-esophagus as a standard TEE probe. In most patients, the first echocardiographic view visualized was the 2-chamber (2C) apical view and the left atrial appendage [53]. Then, thanks to a clockwise rotation, we visualized the interatrial septa, and next we have obtaining both bicaval and aortic short axis views using the anterior-posterior control of the probe. The views obtained were similar to long-axis 110° TEE view of the septum and short-axis 30° TEE view of the septum, respectively. Using these views, we performed TSP. With a counterclockwise rotation, we have obtained the LAA view that we have used for the final device size selection and for device deployment. Technical success was achieved in all patients without peri-procedural complications. Moreover, at the 45 days follow-up no device-related complications (late device embolization, pericardial effusions, thrombus, peri-device leak) occurred. Oursingle-centerr experience suggests that ICE-TEE could be a good alternative imaging technique to guide LAAC procedures without general anesthesia. A trial is ongoing in our cath-lab to define the safety and efficacy of this technique in all patients referred to LAAC and not only in patients with oropharyngeal disease or complex patients in which general anesthesia and TEE carry a non-negligible risk of major complications.

## 7. Conclusions

LAAC currently represents a safe and effective intervention to prevent stroke in AF patients with contraindications to oral anticoagulation or unwillingness to adhere to therapy. Pre-procedural imaging is of primary importance in improving outcomes and should always be carried out either with TEE or with CCTA in order to provide correct sizing measurements and rule out auricular thrombosis. Periprocedural imaging should not be carried out by fluoroscopy alone but should always be accompanied by either TEE or ICE. Even though TEE still remains the gold standard in many laboratories, ICE proved to be safe and effective and can be considered a valuable alternative. TEE-ICE is a new technique, already validated as guidance for other structural heart disease procedures, and clinical trials are needed to validate this interesting imaging technique to guide LAAC. More research is needed in order to move toward a minimalistic approach, aiming to maintain high rates of procedural success while avoiding general anesthesia.

## Data Availability

Not applicable.
